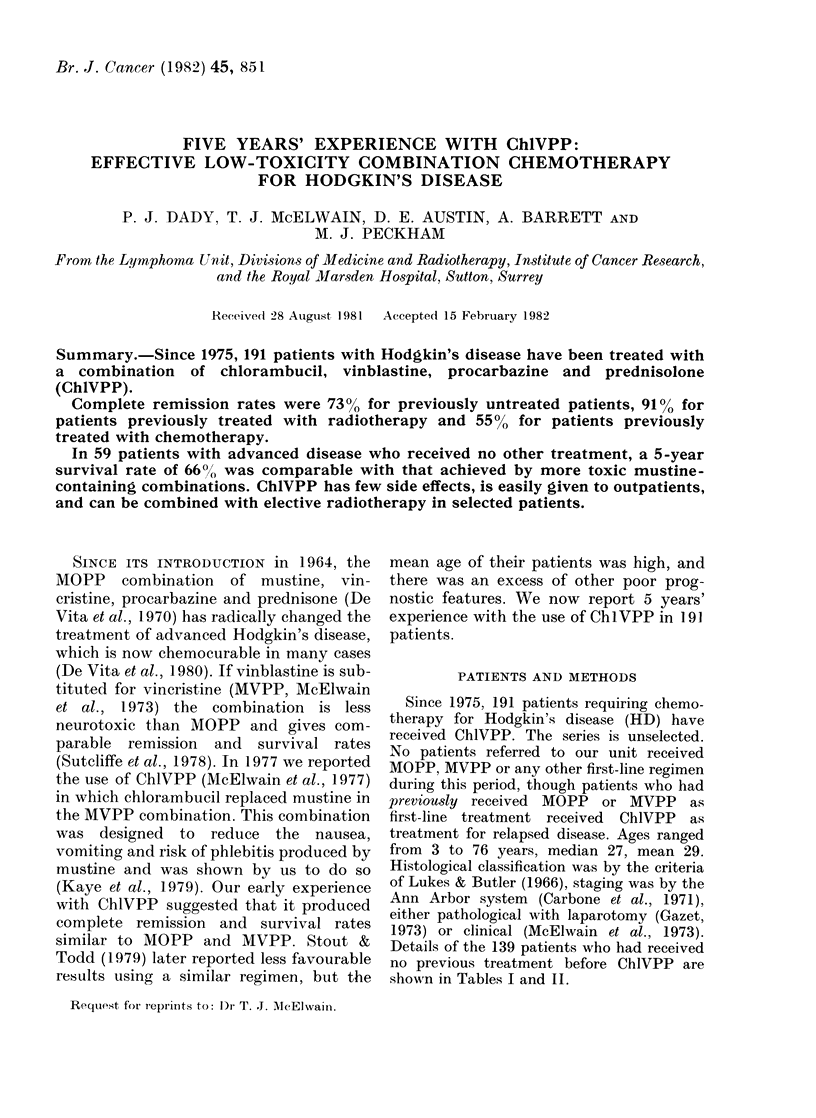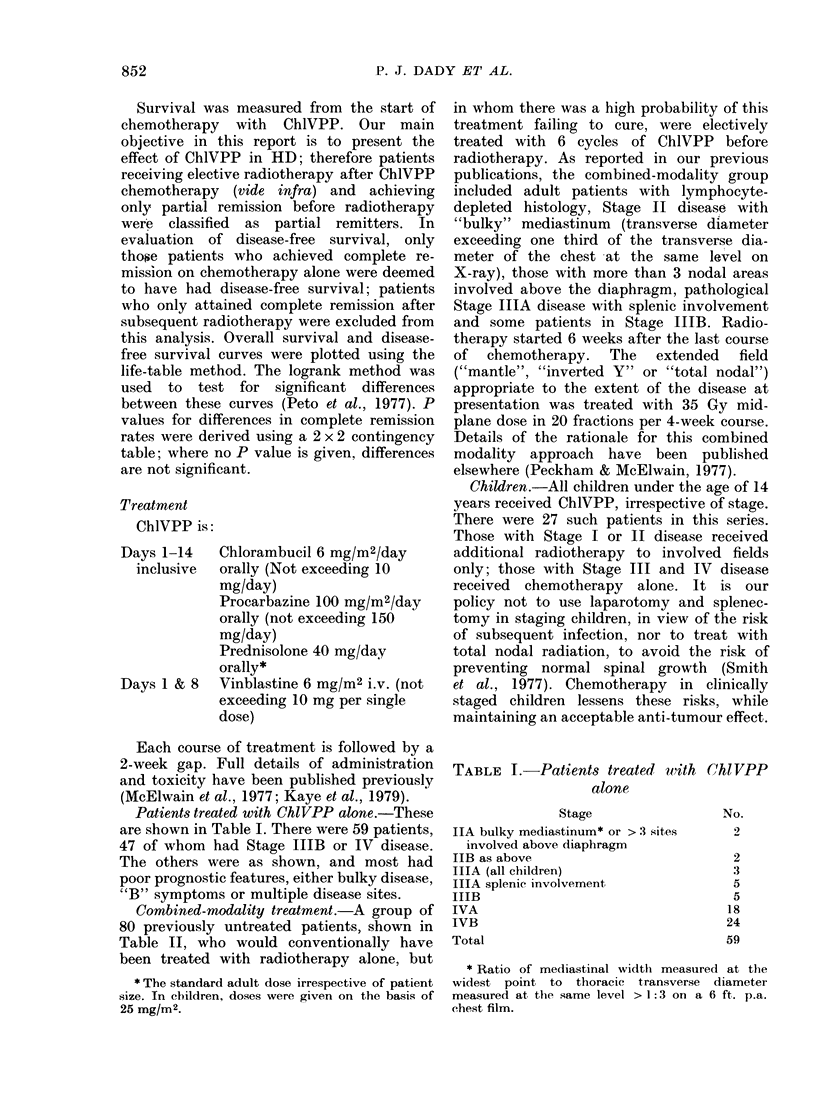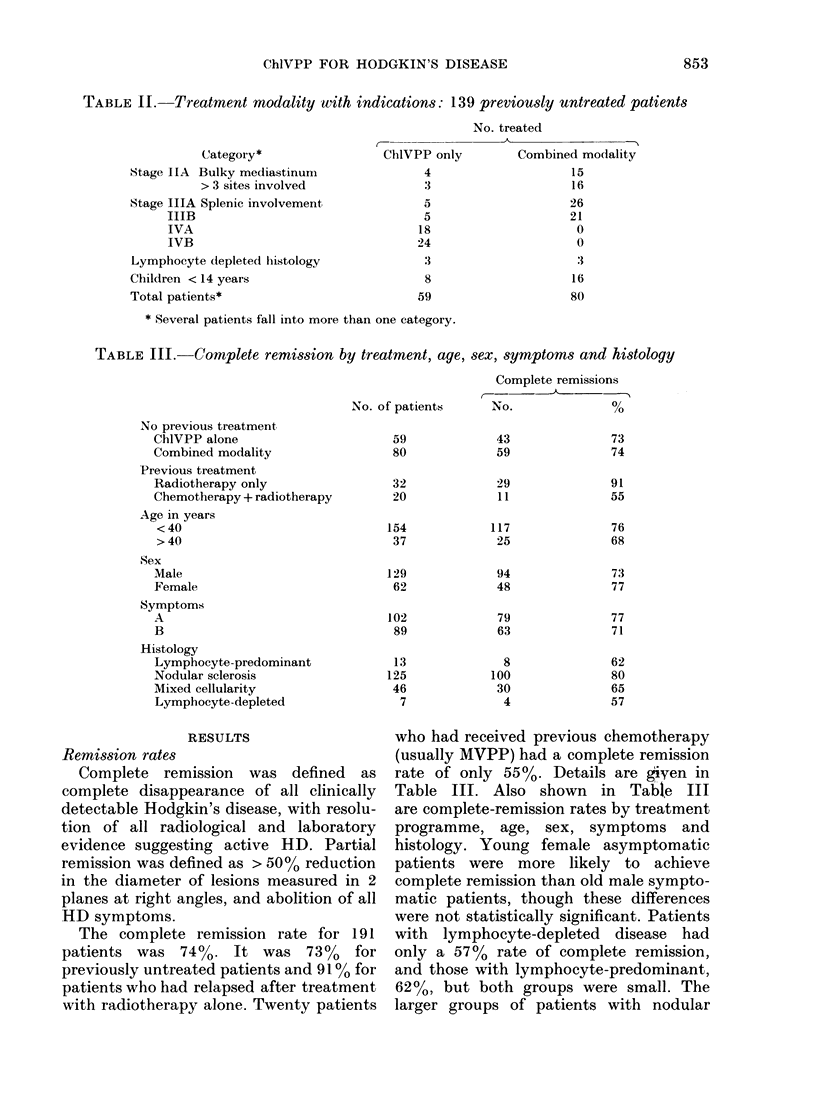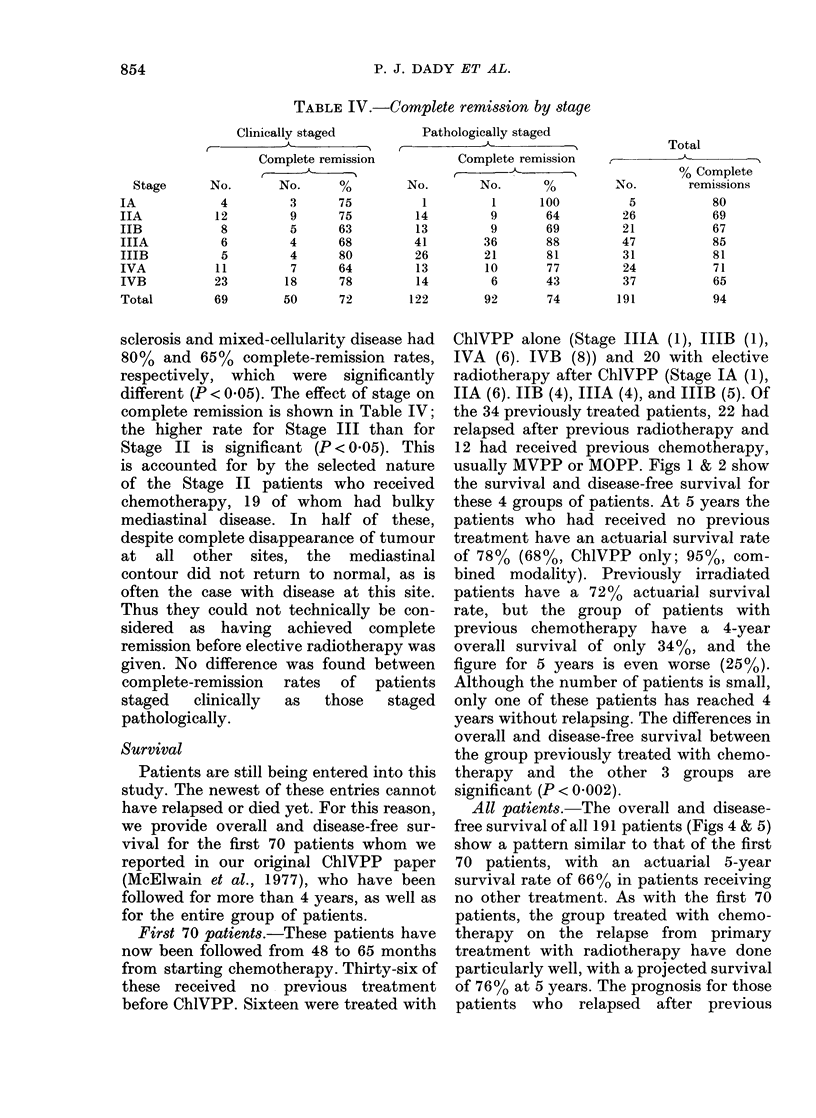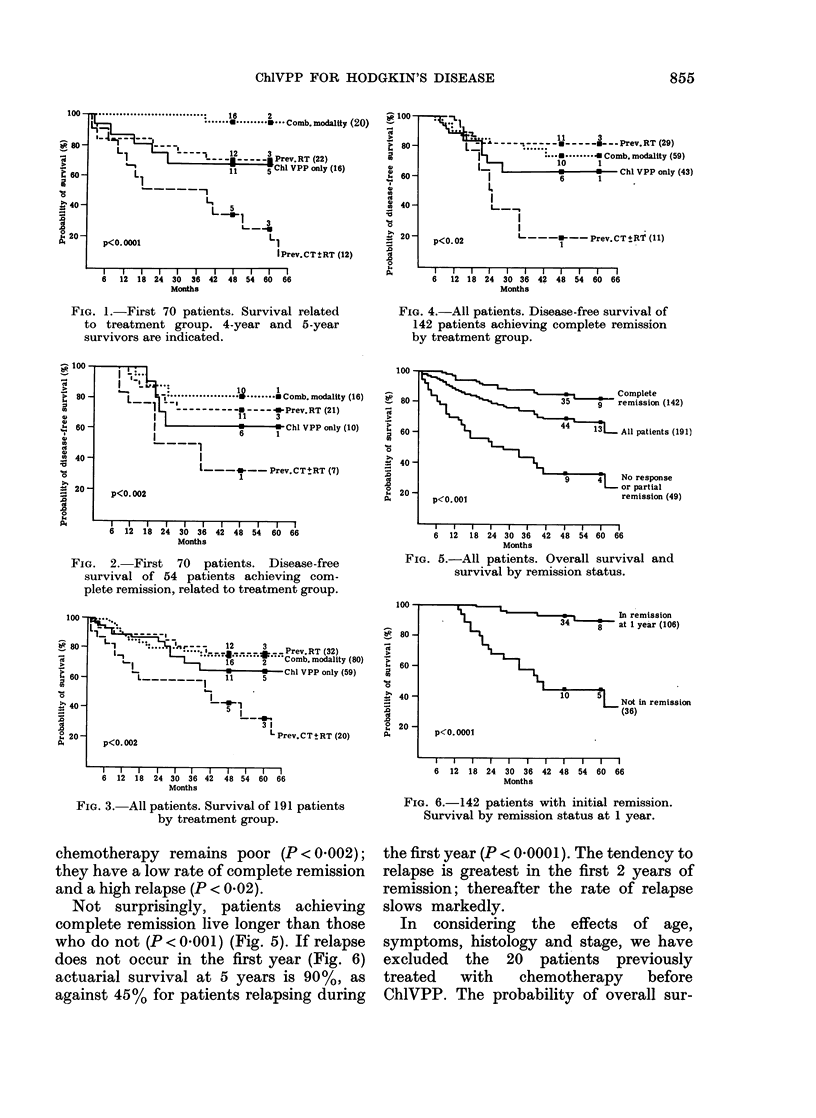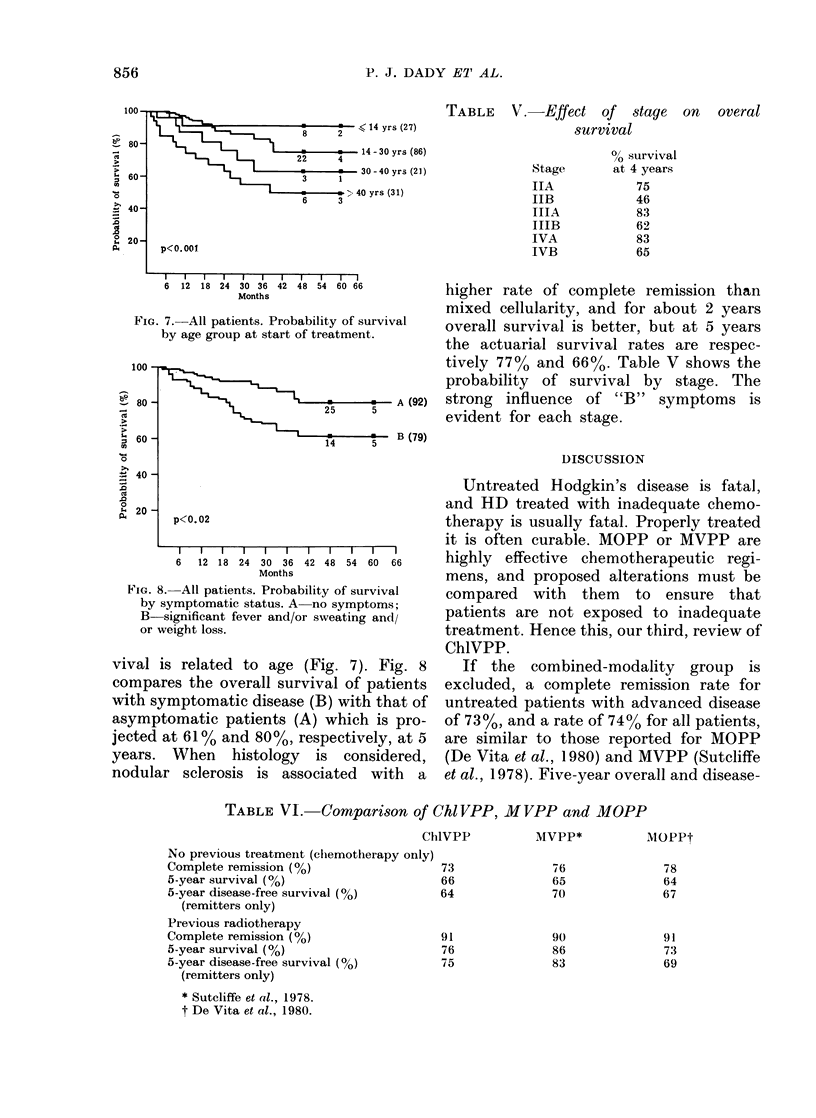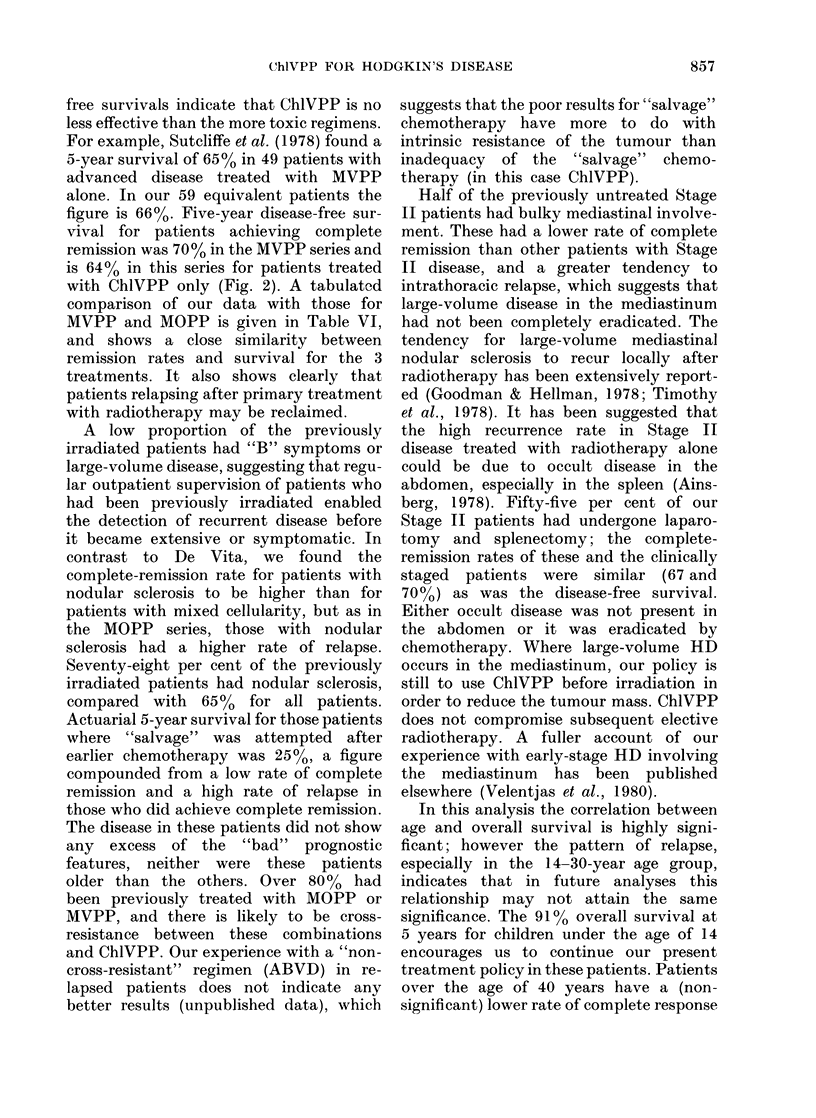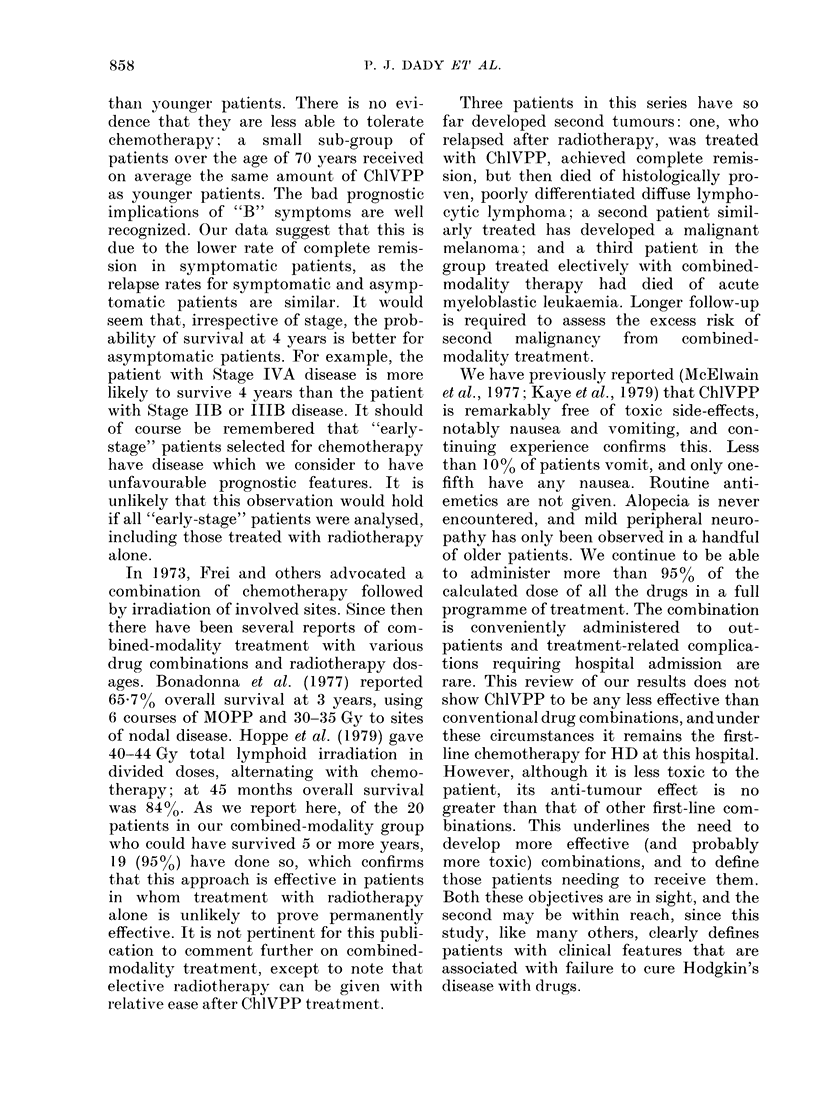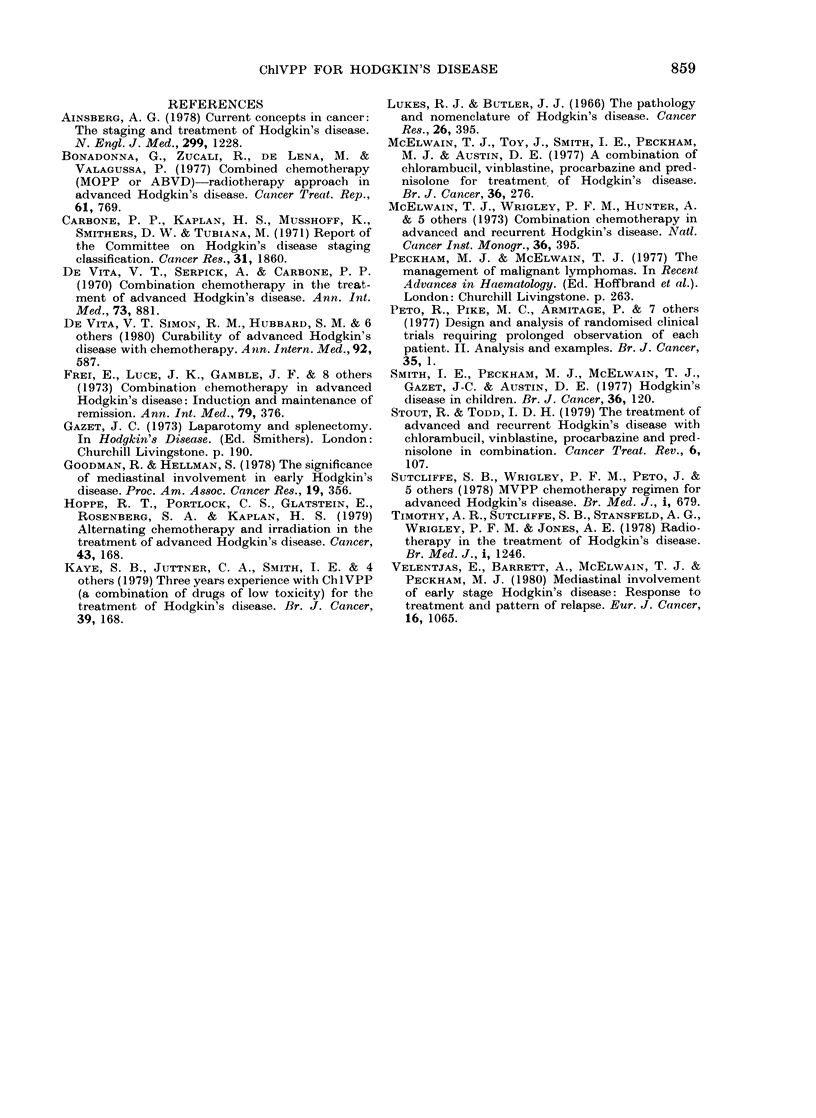# Five years' experience with ChlVPP: effective low-toxicity combination chemotherapy for Hodgkin's disease.

**DOI:** 10.1038/bjc.1982.137

**Published:** 1982-06

**Authors:** P. J. Dady, T. J. McElwain, D. E. Austin, A. Barrett, M. J. Peckham

## Abstract

Since 1975, 191 patients with Hodgkin's disease have been treated with a combination of chlorambucil, vinblastine, procarbazine and prednisolone (ChlVPP). Complete remission rates were 73% for previously untreated patients, 91% for patients previously treated with radiotherapy and 55% for patients previously treated with chemotherapy. In 59 patients with advanced disease who received no other treatment, a 5-year survival rate of 66% was comparable with that achieved by more toxic mustine-containing combinations. ChlVPP has few side effects, is easily given to outpatients, and can be combined with elective radiotherapy in selected patients.


*Br. J. Cancer* (1982) **45**, 851

### FIVE YEARS' EXPERIENCE WITH ChlVPP: EFFECTIVE LOW-TOXICITY COMBINATION CHEMOTHERAPY FOR HODGKIN'S DISEASE

P. J. DADY, T. J. McELWAIN, D. E. AUSTIN, A. BARRETT AND M. J. PECKHAM

*From the Lymphoma Unit, Divisions of Medicine and Radiotherapy, Institute of Cancer Research, and the Royal Marsden Hospital, Sutton, Surrey*

Received 28 August 1981 Accepted 15 February 1982

**Summary.—Since 1975, 191 patients with Hodgkin's disease have been treated with a combination of chlorambucil, vinblastine, procarbazine and prednisolone (ChlVPP).**

**Complete remission rates were 73% for previously untreated patients, 91% for patients previously treated with radiotherapy and 55% for patients previously treated with chemotherapy.**

**In 59 patients with advanced disease who received no other treatment, a 5-year survival rate of 66% was comparable with that achieved by more toxic mustine-containing combinations. ChlVPP has few side effects, is easily given to outpatients, and can be combined with elective radiotherapy in selected patients.**

SINCE ITS INTRODUCTION in 1964, the MOPP combination of mustine, vincristine, procarbazine and prednisone (De Vita *et al.*, 1970) has radically changed the treatment of advanced Hodgkin's disease, which is now chemocurable in many cases (De Vita *et al.*, 1980). If vinblastine is substituted for vincristine (MVPP, McElwain *et al.*, 1973) the combination is less neurotoxic than MOPP and gives comparable remission and survival rates (Sutcliffe *et al.*, 1978). In 1977 we reported the use of ChlVPP (McElwain *et al.*, 1977) in which chlorambucil replaced mustine in the MVPP combination. This combination was designed to reduce the nausea, vomiting and risk of phlebitis produced by mustine and was shown by us to do so (Kaye *et al.*, 1979). Our early experience with ChlVPP suggested that it produced complete remission and survival rates similar to MOPP and MVPP. Stout & Todd (1979) later reported less favourable results using a similar regimen, but the mean age of their patients was high, and there was an excess of other poor prognostic features. We now report 5 years' experience with the use of ChlVPP in 191 patients.

### PATIENTS AND METHODS

Since 1975, 191 patients requiring chemotherapy for Hodgkin's disease (HD) have received ChlVPP. The series is unselected. No patients referred to our unit received MOPP, MVPP or any other first-line regimen during this period, though patients who had *previously* received MOPP or MVPP as first-line treatment received ChlVPP as treatment for relapsed disease. Ages ranged from 3 to 76 years, median 27, mean 29. Histological classification was by the criteria of Lukes & Butler (1966), staging was by the Ann Arbor system (Carbone *et al.*, 1971), either pathological with laparotomy (Gazet, 1973) or clinical (McElwain *et al.*, 1973). Details of the 139 patients who had received no previous treatment before ChlVPP are shown in Tables I and II.

Request for reprints to: Dr T. J. McElwain.

Survival was measured from the start of chemotherapy with ChlVPP. Our main objective in this report is to present the effect of ChlVPP in HD; therefore patients receiving elective radiotherapy after ChlVPP chemotherapy (*vide infra*) and achieving only partial remission before radiotherapy were classified as partial remitters. In evaluation of disease-free survival, only those patients who achieved complete remission on chemotherapy alone were deemed to have had disease-free survival; patients who only attained complete remission after subsequent radiotherapy were excluded from this analysis. Overall survival and disease-free survival curves were plotted using the life-table method. The logrank method was used to test for significant differences between these curves (Peto *et al.*, 1977). $P$ values for differences in complete remission rates were derived using a $2 \times 2$ contingency table; where no $P$ value is given, differences are not significant.

*Treatment*

ChlVPP is:

| | |
|---|---|
| Days 1–14 inclusive | Chlorambucil 6 mg/m²/day orally (Not exceeding 10 mg/day) |
| | Procarbazine 100 mg/m²/day orally (not exceeding 150 mg/day) |
| | Prednisolone 40 mg/day orally* |
| Days 1 & 8 | Vinblastine 6 mg/m² i.v. (not exceeding 10 mg per single dose) |

Each course of treatment is followed by a 2-week gap. Full details of administration and toxicity have been published previously (McElwain *et al.*, 1977; Kaye *et al.*, 1979).

*Patients treated with ChlVPP alone.*—These are shown in Table I. There were 59 patients, 47 of whom had Stage IIIB or IV disease. The others were as shown, and most had poor prognostic features, either bulky disease, "B" symptoms or multiple disease sites.

*Combined-modality treatment.*—A group of 80 previously untreated patients, shown in Table II, who would conventionally have been treated with radiotherapy alone, but in whom there was a high probability of this treatment failing to cure, were electively treated with 6 cycles of ChlVPP before radiotherapy. As reported in our previous publications, the combined-modality group included adult patients with lymphocyte-depleted histology, Stage II disease with "bulky" mediastinum (transverse diameter exceeding one third of the transverse diameter of the chest at the same level on X-ray), those with more than 3 nodal areas involved above the diaphragm, pathological Stage IIIA disease with splenic involvement and some patients in Stage IIIB. Radiotherapy started 6 weeks after the last course of chemotherapy. The extended field ("mantle", "inverted Y" or "total nodal") appropriate to the extent of the disease at presentation was treated with 35 Gy mid-plane dose in 20 fractions per 4-week course. Details of the rationale for this combined modality approach have been published elsewhere (Peckham & McElwain, 1977).

*Children.*—All children under the age of 14 years received ChlVPP, irrespective of stage. There were 27 such patients in this series. Those with Stage I or II disease received additional radiotherapy to involved fields only; those with Stage III and IV disease received chemotherapy alone. It is our policy not to use laparotomy and splenectomy in staging children, in view of the risk of subsequent infection, nor to treat with total nodal radiation, to avoid the risk of preventing normal spinal growth (Smith *et al.*, 1977). Chemotherapy in clinically staged children lessens these risks, while maintaining an acceptable anti-tumour effect.

TABLE I.—*Patients treated with ChlVPP alone*

| Stage | No. |
|---|---|
| IIA bulky mediastinum* or > 3 sites involved above diaphragm | 2 |
| IIB as above | 2 |
| IIIA (all children) | 3 |
| IIIA splenic involvement | 5 |
| IIIB | 5 |
| IVA | 18 |
| IVB | 24 |
| Total | 59 |

* Ratio of mediastinal width measured at the widest point to thoracic transverse diameter measured at the same level > 1:3 on a 6 ft. p.a. chest film.

* The standard adult dose irrespective of patient size. In children, doses were given on the basis of 25 mg/m².

TABLE II.—*Treatment modality with indications: 139 previously untreated patients*

| Category* | No. treated: ChlVPP only | Combined modality |
|---|---|---|
| Stage IIA Bulky mediastinum | 4 | 15 |
| > 3 sites involved | 3 | 16 |
| Stage IIIA Splenic involvement | 5 | 26 |
| IIIB | 5 | 21 |
| IVA | 18 | 0 |
| IVB | 24 | 0 |
| Lymphocyte depleted histology | 3 | 3 |
| Children < 14 years | 8 | 16 |
| Total patients* | 59 | 80 |

* Several patients fall into more than one category.

TABLE III.—*Complete remission by treatment, age, sex, symptoms and histology*

| | No. of patients | Complete remissions: No. | % |
|---|---|---|---|
| No previous treatment | | | |
| ChlVPP alone | 59 | 43 | 73 |
| Combined modality | 80 | 59 | 74 |
| Previous treatment | | | |
| Radiotherapy only | 32 | 29 | 91 |
| Chemotherapy + radiotherapy | 20 | 11 | 55 |
| Age in years | | | |
| < 40 | 154 | 117 | 76 |
| > 40 | 37 | 25 | 68 |
| Sex | | | |
| Male | 129 | 94 | 73 |
| Female | 62 | 48 | 77 |
| Symptoms | | | |
| A | 102 | 79 | 77 |
| B | 89 | 63 | 71 |
| Histology | | | |
| Lymphocyte-predominant | 13 | 8 | 62 |
| Nodular sclerosis | 125 | 100 | 80 |
| Mixed cellularity | 46 | 30 | 65 |
| Lymphocyte-depleted | 7 | 4 | 57 |

### RESULTS

*Remission rates*

Complete remission was defined as complete disappearance of all clinically detectable Hodgkin's disease, with resolution of all radiological and laboratory evidence suggesting active HD. Partial remission was defined as > 50% reduction in the diameter of lesions measured in 2 planes at right angles, and abolition of all HD symptoms.

The complete remission rate for 191 patients was 74%. It was 73% for previously untreated patients and 91% for patients who had relapsed after treatment with radiotherapy alone. Twenty patients who had received previous chemotherapy (usually MVPP) had a complete remission rate of only 55%. Details are given in Table III. Also shown in Table III are complete-remission rates by treatment programme, age, sex, symptoms and histology. Young female asymptomatic patients were more likely to achieve complete remission than old male symptomatic patients, though these differences were not statistically significant. Patients with lymphocyte-depleted disease had only a 57% rate of complete remission, and those with lymphocyte-predominant, 62%, but both groups were small. The larger groups of patients with nodular

TABLE IV.—*Complete remission by stage*

| Stage | Clinically staged No. | Complete remission No. | Complete remission % | Pathologically staged No. | Complete remission No. | Complete remission % | Total No. | Total % Complete remissions |
|---|---|---|---|---|---|---|---|---|
| IA | 4 | 3 | 75 | 1 | 1 | 100 | 5 | 80 |
| IIA | 12 | 9 | 75 | 14 | 9 | 64 | 26 | 69 |
| IIB | 8 | 5 | 63 | 13 | 9 | 69 | 21 | 67 |
| IIIA | 6 | 4 | 68 | 41 | 36 | 88 | 47 | 85 |
| IIIB | 5 | 4 | 80 | 26 | 21 | 81 | 31 | 81 |
| IVA | 11 | 7 | 64 | 13 | 10 | 77 | 24 | 71 |
| IVB | 23 | 18 | 78 | 14 | 6 | 43 | 37 | 65 |
| Total | 69 | 50 | 72 | 122 | 92 | 74 | 191 | 94 |

sclerosis and mixed-cellularity disease had 80% and 65% complete-remission rates, respectively, which were significantly different ($P < 0{\cdot}05$). The effect of stage on complete remission is shown in Table IV; the higher rate for Stage III than for Stage II is significant ($P < 0{\cdot}05$). This is accounted for by the selected nature of the Stage II patients who received chemotherapy, 19 of whom had bulky mediastinal disease. In half of these, despite complete disappearance of tumour at all other sites, the mediastinal contour did not return to normal, as is often the case with disease at this site. Thus they could not technically be considered as having achieved complete remission before elective radiotherapy was given. No difference was found between complete-remission rates of patients staged clinically as those staged pathologically.

*Survival*

Patients are still being entered into this study. The newest of these entries cannot have relapsed or died yet. For this reason, we provide overall and disease-free survival for the first 70 patients whom we reported in our original ChlVPP paper (McElwain *et al.*, 1977), who have been followed for more than 4 years, as well as for the entire group of patients.

*First* 70 *patients*.—These patients have now been followed from 48 to 65 months from starting chemotherapy. Thirty-six of these received no previous treatment before ChlVPP. Sixteen were treated with ChlVPP alone (Stage IIIA (1), IIIB (1), IVA (6). IVB (8)) and 20 with elective radiotherapy after ChlVPP (Stage IA (1), IIA (6). IIB (4), IIIA (4), and IIIB (5). Of the 34 previously treated patients, 22 had relapsed after previous radiotherapy and 12 had received previous chemotherapy, usually MVPP or MOPP. Figs 1 & 2 show the survival and disease-free survival for these 4 groups of patients. At 5 years the patients who had received no previous treatment have an actuarial survival rate of 78% (68%, ChlVPP only; 95%, combined modality). Previously irradiated patients have a 72% actuarial survival rate, but the group of patients with previous chemotherapy have a 4-year overall survival of only 34%, and the figure for 5 years is even worse (25%). Although the number of patients is small, only one of these patients has reached 4 years without relapsing. The differences in overall and disease-free survival between the group previously treated with chemotherapy and the other 3 groups are significant ($P < 0{\cdot}002$).

*All patients*.—The overall and disease-free survival of all 191 patients (Figs 4 & 5) show a pattern similar to that of the first 70 patients, with an actuarial 5-year survival rate of 66% in patients receiving no other treatment. As with the first 70 patients, the group treated with chemotherapy on the relapse from primary treatment with radiotherapy have done particularly well, with a projected survival of 76% at 5 years. The prognosis for those patients who relapsed after previous

FIG. 1.—First 70 patients. Survival related to treatment group. 4-year and 5-year survivors are indicated.

FIG. 2.—First 70 patients. Disease-free survival of 54 patients achieving complete remission, related to treatment group.

FIG. 3.—All patients. Survival of 191 patients by treatment group.

FIG. 4.—All patients. Disease-free survival of 142 patients achieving complete remission by treatment group.

FIG. 5.—All patients. Overall survival and survival by remission status.

FIG. 6.—142 patients with initial remission. Survival by remission status at 1 year.

chemotherapy remains poor ($P < 0{\cdot}002$); they have a low rate of complete remission and a high relapse ($P < 0{\cdot}02$).

Not surprisingly, patients achieving complete remission live longer than those who do not ($P < 0{\cdot}001$) (Fig. 5). If relapse does not occur in the first year (Fig. 6) actuarial survival at 5 years is 90%, as against 45% for patients relapsing during the first year ($P < 0{\cdot}0001$). The tendency to relapse is greatest in the first 2 years of remission; thereafter the rate of relapse slows markedly.

In considering the effects of age, symptoms, histology and stage, we have excluded the 20 patients previously treated with chemotherapy before ChlVPP. The probability of overall sur-

Fig. 7.—All patients. Probability of survival by age group at start of treatment.

Fig. 8.—All patients. Probability of survival by symptomatic status. A—no symptoms; B—significant fever and/or sweating and/or weight loss.

vival is related to age (Fig. 7). Fig. 8 compares the overall survival of patients with symptomatic disease (B) with that of asymptomatic patients (A) which is projected at 61% and 80%, respectively, at 5 years. When histology is considered, nodular sclerosis is associated with a higher rate of complete remission than mixed cellularity, and for about 2 years overall survival is better, but at 5 years the actuarial survival rates are respectively 77% and 66%. Table V shows the probability of survival by stage. The strong influence of "B" symptoms is evident for each stage.

Table V.—*Effect of stage on overal survival*

| Stage | % survival at 4 years |
|---|---|
| IIA | 75 |
| IIB | 46 |
| IIIA | 83 |
| IIIB | 62 |
| IVA | 83 |
| IVB | 65 |

### DISCUSSION

Untreated Hodgkin's disease is fatal, and HD treated with inadequate chemotherapy is usually fatal. Properly treated it is often curable. MOPP or MVPP are highly effective chemotherapeutic regimens, and proposed alterations must be compared with them to ensure that patients are not exposed to inadequate treatment. Hence this, our third, review of ChlVPP.

If the combined-modality group is excluded, a complete remission rate for untreated patients with advanced disease of 73%, and a rate of 74% for all patients, are similar to those reported for MOPP (De Vita *et al.*, 1980) and MVPP (Sutcliffe *et al.*, 1978). Five-year overall and disease-

Table VI.—*Comparison of ChlVPP, MVPP and MOPP*

| | ChlVPP | MVPP* | MOPP† |
|---|---|---|---|
| No previous treatment (chemotherapy only) | | | |
| Complete remission (%) | 73 | 76 | 78 |
| 5-year survival (%) | 66 | 65 | 64 |
| 5-year disease-free survival (%) (remitters only) | 64 | 70 | 67 |
| Previous radiotherapy | | | |
| Complete remission (%) | 91 | 90 | 91 |
| 5-year survival (%) | 76 | 86 | 73 |
| 5-year disease-free survival (%) (remitters only) | 75 | 83 | 69 |

* Sutcliffe *et al.*, 1978.

† De Vita *et al.*, 1980.

free survivals indicate that ChlVPP is no less effective than the more toxic regimens. For example, Sutcliffe *et al.* (1978) found a 5-year survival of 65% in 49 patients with advanced disease treated with MVPP alone. In our 59 equivalent patients the figure is 66%. Five-year disease-free survival for patients achieving complete remission was 70% in the MVPP series and is 64% in this series for patients treated with ChlVPP only (Fig. 2). A tabulated comparison of our data with those for MVPP and MOPP is given in Table VI, and shows a close similarity between remission rates and survival for the 3 treatments. It also shows clearly that patients relapsing after primary treatment with radiotherapy may be reclaimed.

A low proportion of the previously irradiated patients had "B" symptoms or large-volume disease, suggesting that regular outpatient supervision of patients who had been previously irradiated enabled the detection of recurrent disease before it became extensive or symptomatic. In contrast to De Vita, we found the complete-remission rate for patients with nodular sclerosis to be higher than for patients with mixed cellularity, but as in the MOPP series, those with nodular sclerosis had a higher rate of relapse. Seventy-eight per cent of the previously irradiated patients had nodular sclerosis, compared with 65% for all patients. Actuarial 5-year survival for those patients where "salvage" was attempted after earlier chemotherapy was 25%, a figure compounded from a low rate of complete remission and a high rate of relapse in those who did achieve complete remission. The disease in these patients did not show any excess of the "bad" prognostic features, neither were these patients older than the others. Over 80% had been previously treated with MOPP or MVPP, and there is likely to be cross-resistance between these combinations and ChlVPP. Our experience with a "non-cross-resistant" regimen (ABVD) in relapsed patients does not indicate any better results (unpublished data), which suggests that the poor results for "salvage" chemotherapy have more to do with intrinsic resistance of the tumour than inadequacy of the "salvage" chemotherapy (in this case ChlVPP).

Half of the previously untreated Stage II patients had bulky mediastinal involvement. These had a lower rate of complete remission than other patients with Stage II disease, and a greater tendency to intrathoracic relapse, which suggests that large-volume disease in the mediastinum had not been completely eradicated. The tendency for large-volume mediastinal nodular sclerosis to recur locally after radiotherapy has been extensively reported (Goodman & Hellman, 1978; Timothy *et al.*, 1978). It has been suggested that the high recurrence rate in Stage II disease treated with radiotherapy alone could be due to occult disease in the abdomen, especially in the spleen (Ainsberg, 1978). Fifty-five per cent of our Stage II patients had undergone laparotomy and splenectomy; the complete-remission rates of these and the clinically staged patients were similar (67 and 70%) as was the disease-free survival. Either occult disease was not present in the abdomen or it was eradicated by chemotherapy. Where large-volume HD occurs in the mediastinum, our policy is still to use ChlVPP before irradiation in order to reduce the tumour mass. ChlVPP does not compromise subsequent elective radiotherapy. A fuller account of our experience with early-stage HD involving the mediastinum has been published elsewhere (Velentjas *et al.*, 1980).

In this analysis the correlation between age and overall survival is highly significant; however the pattern of relapse, especially in the 14–30-year age group, indicates that in future analyses this relationship may not attain the same significance. The 91% overall survival at 5 years for children under the age of 14 encourages us to continue our present treatment policy in these patients. Patients over the age of 40 years have a (non-significant) lower rate of complete response than younger patients. There is no evidence that they are less able to tolerate chemotherapy; a small sub-group of patients over the age of 70 years received on average the same amount of ChlVPP as younger patients. The bad prognostic implications of "B" symptoms are well recognized. Our data suggest that this is due to the lower rate of complete remission in symptomatic patients, as the relapse rates for symptomatic and asymptomatic patients are similar. It would seem that, irrespective of stage, the probability of survival at 4 years is better for asymptomatic patients. For example, the patient with Stage IVA disease is more likely to survive 4 years than the patient with Stage IIB or IIIB disease. It should of course be remembered that "early-stage" patients selected for chemotherapy have disease which we consider to have unfavourable prognostic features. It is unlikely that this observation would hold if all "early-stage" patients were analysed, including those treated with radiotherapy alone.

In 1973, Frei and others advocated a combination of chemotherapy followed by irradiation of involved sites. Since then there have been several reports of combined-modality treatment with various drug combinations and radiotherapy dosages. Bonadonna *et al.* (1977) reported 65·7% overall survival at 3 years, using 6 courses of MOPP and 30–35 Gy to sites of nodal disease. Hoppe *et al.* (1979) gave 40–44 Gy total lymphoid irradiation in divided doses, alternating with chemotherapy; at 45 months overall survival was 84%. As we report here, of the 20 patients in our combined-modality group who could have survived 5 or more years, 19 (95%) have done so, which confirms that this approach is effective in patients in whom treatment with radiotherapy alone is unlikely to prove permanently effective. It is not pertinent for this publication to comment further on combined-modality treatment, except to note that elective radiotherapy can be given with relative ease after ChlVPP treatment.

Three patients in this series have so far developed second tumours: one, who relapsed after radiotherapy, was treated with ChlVPP, achieved complete remission, but then died of histologically proven, poorly differentiated diffuse lymphocytic lymphoma; a second patient similarly treated has developed a malignant melanoma; and a third patient in the group treated electively with combined-modality therapy had died of acute myeloblastic leukaemia. Longer follow-up is required to assess the excess risk of second malignancy from combined-modality treatment.

We have previously reported (McElwain *et al.*, 1977; Kaye *et al.*, 1979) that ChlVPP is remarkably free of toxic side-effects, notably nausea and vomiting, and continuing experience confirms this. Less than 10% of patients vomit, and only one-fifth have any nausea. Routine anti-emetics are not given. Alopecia is never encountered, and mild peripheral neuropathy has only been observed in a handful of older patients. We continue to be able to administer more than 95% of the calculated dose of all the drugs in a full programme of treatment. The combination is conveniently administered to out-patients and treatment-related complications requiring hospital admission are rare. This review of our results does not show ChlVPP to be any less effective than conventional drug combinations, and under these circumstances it remains the first-line chemotherapy for HD at this hospital. However, although it is less toxic to the patient, its anti-tumour effect is no greater than that of other first-line combinations. This underlines the need to develop more effective (and probably more toxic) combinations, and to define those patients needing to receive them. Both these objectives are in sight, and the second may be within reach, since this study, like many others, clearly defines patients with clinical features that are associated with failure to cure Hodgkin's disease with drugs.

### REFERENCES

AINSBERG, A. G. (1978) Current concepts in cancer: The staging and treatment of Hodgkin's disease. *N. Engl. J. Med.*, **299**, 1228.

BONADONNA, G., ZUCALI, R., DE LENA, M. & VALAGUSSA, P. (1977) Combined chemotherapy (MOPP or ABVD)—radiotherapy approach in advanced Hodgkin's disease. *Cancer Treat. Rep.*, **61**, 769.

CARBONE, P. P., KAPLAN, H. S., MUSSHOFF, K., SMITHERS, D. W. & TUBIANA, M. (1971) Report of the Committee on Hodgkin's disease staging classification. *Cancer Res.*, **31**, 1860.

DE VITA, V. T., SERPICK, A. & CARBONE, P. P. (1970) Combination chemotherapy in the treatment of advanced Hodgkin's disease. *Ann. Int. Med.*, **73**, 881.

DE VITA, V. T. SIMON, R. M., HUBBARD, S. M. & 6 others (1980) Curability of advanced Hodgkin's disease with chemotherapy. *Ann. Intern. Med.*, **92**, 587.

FREI, E., LUCE, J. K., GAMBLE, J. F. & 8 others (1973) Combination chemotherapy in advanced Hodgkin's disease: Induction and maintenance of remission. *Ann. Int. Med.*, **79**, 376.

GAZET, J. C. (1973) Laparotomy and splenectomy. In *Hodgkin's Disease.* (Ed. Smithers). London: Churchill Livingstone. p. 190.

GOODMAN, R. & HELLMAN, S. (1978) The significance of mediastinal involvement in early Hodgkin's disease. *Proc. Am. Assoc. Cancer Res.*, **19**, 356.

HOPPE, R. T., PORTLOCK, C. S., GLATSTEIN, E., ROSENBERG, S. A. & KAPLAN, H. S. (1979) Alternating chemotherapy and irradiation in the treatment of advanced Hodgkin's disease. *Cancer*, **43**, 168.

KAYE, S. B., JUTTNER, C. A., SMITH, I. E. & 4 others (1979) Three years experience with ChlVPP (a combination of drugs of low toxicity) for the treatment of Hodgkin's disease. *Br. J. Cancer*, **39**, 168.

LUKES, R. J. & BUTLER, J. J. (1966) The pathology and nomenclature of Hodgkin's disease. *Cancer Res.*, **26**, 395.

McELWAIN, T. J., TOY, J., SMITH, I. E., PECKHAM, M. J. & AUSTIN, D. E. (1977) A combination of chlorambucil, vinblastine, procarbazine and prednisolone for treatment of Hodgkin's disease. *Br. J. Cancer*, **36**, 276.

McELWAIN, T. J., WRIGLEY, P. F. M., HUNTER, A. & 5 others (1973) Combination chemotherapy in advanced and recurrent Hodgkin's disease. *Natl. Cancer Inst. Monogr.*, **36**, 395.

PECKHAM, M. J. & McELWAIN, T. J. (1977) The management of malignant lymphomas. In *Recent Advances in Haematology.* (Ed. Hoffbrand *et al.*). London: Churchill Livingstone. p. 263.

PETO, R., PIKE, M. C., ARMITAGE, P. & 7 others (1977) Design and analysis of randomised clinical trials requiring prolonged observation of each patient. II. Analysis and examples. *Br. J. Cancer*, **35**, 1.

SMITH, I. E., PECKHAM, M. J., McELWAIN, T. J., GAZET, J-C. & AUSTIN, D. E. (1977) Hodgkin's disease in children. *Br. J. Cancer*, **36**, 120.

STOUT, R. & TODD, I. D. H. (1979) The treatment of advanced and recurrent Hodgkin's disease with chlorambucil, vinblastine, procarbazine and prednisolone in combination. *Cancer Treat. Rev.*, **6**, 107.

SUTCLIFFE, S. B., WRIGLEY, P. F. M., PETO, J. & 5 others (1978) MVPP chemotherapy regimen for advanced Hodgkin's disease. *Br. Med. J.*, **i**, 679.

TIMOTHY, A. R., SUTCLIFFE, S. B., STANSFELD, A. G., WRIGLEY, P. F. M. & JONES, A. E. (1978) Radiotherapy in the treatment of Hodgkin's disease. *Br. Med. J.*, **i**, 1246.

VELENTJAS, E., BARRETT, A., McELWAIN, T. J. & PECKHAM, M. J. (1980) Mediastinal involvement of early stage Hodgkin's disease: Response to treatment and pattern of relapse. *Eur. J. Cancer*, **16**, 1065.